# Effect of Digestion on Ursolic Acid Self-Stabilized Water-in-Oil Emulsion: Role of Bile Salts

**DOI:** 10.3390/foods12193657

**Published:** 2023-10-03

**Authors:** Yumeng Yan, Yugang Liu, Chaoxi Zeng, Huiping Xia

**Affiliations:** 1 Department of Food Science and Technology, College of Food Science and Technology, Hunan Agricultural University, No.1 Nongda Road, Furong District, Changsha 410128, China; feyanym@mail.scut.edu.cn (Y.Y.); gang@stu.hunau.edu.cn (Y.L.); chaoxizeng@hunau.edu.cn (C.Z.); 2 Department of Food Science and Technology, College of Food Science and Technology, South China University of Technology, Guangzhou 510640, China

**Keywords:** ursolic acid emulsion, bile salt concentration, in vitro simulated digestion

## Abstract

Exploring the effect of bile salts on the properties of emulsion carriers containing hydrophobic bioactive compounds is particularly critical to understanding the stability and bioavailability of these hydrophobic bioactive compounds in the digestive process. In this study, the effects of bile salts on the stability and digestive characteristics of the ursolic acid (UA) self-stabilized water-in-oil (W/O) emulsion were investigated via static and dynamic (with or without enzyme) in vitro simulated digestive systems. The results showed that under the static system, the basic conditions had less interference, while the bile salts had a significant effect on the appearance and microstructure of the emulsion. The primary mechanism of emulsion instability is hydrophobic binding and depletion flocculation. Under the dynamic condition, it was found that the low concentrations of bile salts can promote the release amount and the rate of free fatty acids via displacement, while high concentrations of bile salts inhibit the decomposition of lipid, which may be related to the secondary coverage formed at the interface by the bile salts. These findings provide a theoretical basis for understanding the digestive behavior of the UA emulsion and its interaction with bile salts, which are conducive to developing and designing new emulsions to improve the bioaccessibility of UA.

## 1. Introduction

Pentacyclic triterpenoids are widely found in various plants. They are one of the active ingredients of many medicinal materials, which have rich biological activities, mainly reflected in liver protection and the regulation of glucose and lipid metabolism disorders [1]. In recent years, its anti-inflammatory, antioxidant, antibacterial, anticancer, and other pharmacological effects have also attracted attention [2,3], which is a hotspot in medicine and health food. However, due to the polycyclic alkane structure, the water solubility of pentacyclic triterpenes is generally poor and the oral bioavailability is very low, which seriously limits the practical application of these compounds. Numerous experiments have been conducted on how to improve its biological accessibility [4,5,6]. Bioaccessibility refers to the proportion of substances released from the food matrix after digestion in the gastrointestinal tract that can be absorbed and utilized by the body [7]. It is influenced by the interaction between the bioactive substances and the digestive fluid, as well as by different types of carriers. Many studies have demonstrated that the lipid-based delivery system has a significant absorption-enhancing effect on most hydrophobic bioactive compounds [8,9].

An emulsion is a thermodynamically unstable dispersed system formed by dispersing one liquid in the form of tiny droplets (usually about 0.1~10 μm in diameter) into another immiscible liquid [10]. It is an important food form and can be divided into the oil-in-water (O/W) emulsion, the water-in-oil (W/O) emulsion, and multiple emulsions according to the difference in the continuous phase. Some studies have found that the emulsion form positively affects the bioavailability of bioactive compounds. The construction of a nanoemulsion delivery system (NDS) or nanoemulsion excipient system (NES) can improve the oral availability of bioactive compounds in different ranges [11]. The emulsion composition and the mode of delivery in contact with the bioactive compounds cause different gastrointestinal reactions, in which the rapid digestion of the lipid phase results in the formation of mixed micelles capable of dissolving and transporting hydrophobic bioactive compounds [12]. Secondly, the addition of bioactive compounds to the lipid droplets protects them from the chemical degradation caused by the components in the aqueous phase [13]. In addition, certain lipid digests can increase the permeability of epithelial cell membranes, thereby promoting the co-absorption of bioactive compounds with lipolytic products [14]. In recent years, self-emulsifying drug delivery systems (SEDDS) have also been commonly used to encapsulate lipophilic active molecules in various foods and drugs, such as curcumin, dihydromyricetin, silybin, etc., which can improve their solubility and bioavailability [15].

Only limited lipohydrolysis occurs in the oral and gastric phases of the emulsion [16]. When partially digested emulsions enter the small intestine from the stomach, they are further exposed to complex physicochemical conditions, among which bile salt is an important influencing factor [17]. It is a sodium or potassium salt formed by combining the bile acids secreted by liver cells with glycine or taurine. Recent studies have shown that bile salts can participate in lipid digestion via the following mechanisms: (1) Reduces the surface tension and emulsifies the lipid droplets, thereby increasing the contact area between lipase and lipids. (2) Accelerates the adsorption of lipase via displacement [18]. (3) Together with lipolysis products, hybrid micelles are formed, aiding their transport and absorption [19,20]. (4) The dynamic adsorption and desorption behavior can coordinate with lipolysis in real time. (5) Destroys the network structure of intestinal salt ions, such as Ca^2+^ and short-chain fatty acids, to promote lipolysis [21]. However, there are few studies on the interaction between the emulsion system and digestive fluid, and most of the experiments focus on the O/W emulsion. People know little about the interaction between the W/O emulsion and digestive fluid, especially about the role of bile salts in the digestive process of the W/O emulsion.

Previous studies have found that typical pentacyclic triterpenoids such as ursolic acid and oleanolic acid have self-assembly characteristics, can form stable W/O emulsions, and their bioaccessibility is significantly improved compared with direct oral administration [22,23]. It shows that the emulsion form can solve the problem of the low bioavailability of pentacyclic triterpenoids. Therefore, this study prepared a ursolic acid (UA) self-stabilized W/O emulsion system for carrying this typical pentacyclic triterpene. For the first time, the behaviors of the UA W/O Pickering emulsion during digestion and the impact mechanism of bile salts on it were explored. We first ignored the processing of the emulsion in the oral cavity and stomach and established a static in vitro simulated small intestinal digestion model (without pancreatic enzymes) to elucidate the effects of bile salts on the properties of the emulsion by monitoring the changes of appearance and morphology, microstructure, particle size, and the potential of the emulsion. Then, a dynamic oral–gastroenteric digestive system was established to measure the release amount and the rate of free fatty acids, as well as the UA content in the micelle phase of the digestive fluid. Understanding the digestion of the UA emulsion in the human body and its interaction with bile salts provides new research ideas for the full utilization of UA and other bioactive compounds, and it also provides a theoretical basis for expanding the application of the emulsion for the delivery of nutrients and medicines.

## 2. Materials and Methods

### 2.1. Materials

Ursolic Acid (UA, ≥98%) in the form of a white powder was purchased from Shaanxi Jin Kang Tai Biological Technology Co., Ltd. (Xi Xian New Area, China). UA was used without further purification. Rapeseed oil was purchased from a local store. Bile salt, pepsin (≥250 U/mg), pancreatic enzyme (P7545, 8 USP), and lipase (L3126, from porcine pancreas type II, 100–500 units/mg protein) were obtained from Sigma Chemical Company. Phosphate buffered saline (PBS, pH 7.4) was obtained from Gibco Life Technologies (Grand Island, NY, USA). All other reagents were of analytical grade.

### 2.2. Preparation of W/O Emulsion

UA particles (oil–water ratio = 7:3, 3 wt% oil) were dispersed in rapeseed oil, and the UA oil dispersion was prepared via continuous stirring in a constant temperature water/oil bath at 80 °C for 20 min. The 30 wt% aqueous phase was homogenized with the UA oil dispersion in a T18 digital high-speed disperser Ultra-Turrax (IKA, Staufen, Germany) for 2 min at 13,600 rpm to produce the W/O emulsion, which was sealed and stored under refrigeration immediately after preparation.

### 2.3. In Vitro Static Simulation of Intestinal Digestion

Synthesizing the methods of Qian [24], Sarkar [25], and Zhan [26] et al., with minor modifications, the simulated intestinal fluid (SIF) is composed of bile extracts, intestinal salts, pancreatin, and lipase composition. An amount of 0.1755 g of NaCl was weighed and mixed with 10 mL of distilled water in a clean beaker, and a 1.0 mL CaCl_2_ solution (containing 110 mg of CaCl_2_) and 7.6 mL of a 0.1 mol/L NaOH solution were added. Different concentrations of bile salts were selected according to the experiment, pre-dissolved with PBS, stirred, and added to the system, and the pH was adjusted to the desired value (6.5 or 7.0). The prepared SIF should be preheated at 95 °C for 5 min to exclude any possible enzyme activity in the bile salts. After the SIF is cooled, 1 g of the UA emulsion is added and the resulting mixture is incubated for 2 h at 37 °C and 600 rpm.

### 2.4. Polarized Light Microscopy

The optical microstructures of the original emulsion, emulsion–water, emulsion–enteric salt, and emulsion–SIF mixture were observed using a BX53M polarized light microscope (Olympus, Tokyo, Japan). A drop of the upper emulsion was taken on a slide, covered with a coverslip, and compacted. Then, the structures were observed with 10×, 20×, and 50× objectives and photographed and recorded, respectively.

### 2.5. Measurement of Droplet Size

A plain microscopic image of the emulsion was obtained via a 50× lens of a polarized light microscope, and the emulsion (~2 μL) was deposited on a slide and covered with a coverslip. The average surface diameter of the emulsion (D_(3,2)_) was estimated using Nano Measure 1.2 software and 100 droplets were selected for counting. D_(3,2)_ was defined as:

(1) $$D_{\left(3,2\right)}=\frac{\sum_{nidi}3}{\sum_{nidi}2}$$

### 2.6. ζ-Potential Measurements

The emulsion–SIF mixture and the aqueous phase after centrifugation of the mixture were diluted to approximately a 0.005 wt% droplet concentration, choosing an appropriate background buffer solution and using a nanoparticle sizer ZETASIZER NANO ZS (Malvern, UK) before being analyzed via laser Doppler velocimetry and the phase light scattering (M3-PALS) technique, to measure the ζ-potential of the sample. Each individual ζ-potential data point was calculated from the mean and standard deviation of at least ten readings on the same sample.

### 2.7. In Vitro Dynamic Oral–Gastrointestinal Simulation of Digestion

#### 2.7.1. Oral Phase

According to Ojeda-Serna [27] et al., with slight modification, 1 g of the emulsion was mixed with 9 mL of the physiological salt solution and mucin (30 mg/mL), 25 μL of 0.3 M CaCl_2_, 975 μL of distilled water, and a final volume of 10 mL, and then incubated for 2 min at 37 °C.

#### 2.7.2. Gastric Phase

SGF was prepared by mixing 10 mL of the oral phase with 10 mL of the NaCl solution (2 g/L) and adding 1.0 M of HCl to adjust the pH to 2.0 [28]. Porcine pepsin (3.2 mg/mL) was added and dispersed in the SGF. The pH of the mixture was adjusted to 2.0 and incubated at 37 °C with constant stirring (100 rpm) for 2 h.

#### 2.7.3. Intestinal Phase

According to the method of Qian et al. [24], with slight modifications, SIF consists of bile extract, saline solution, pancreatin, and lipase. Take the stomach sample (20 mL) in a clean beaker, incubate it in a water bath (37 °C) for 10 min, and then adjust the pH to 7 with the NaOH solution (0.05–1 M). Bile salts of different concentrations were selected according to the experiment, pre-dissolved in PBS, and added to 20 mL of chyme with stirring to adjust the pH to 6.8. Then, 1.0 mL of the CaCl_2_ solution (containing 110 mg of CaCl_2_) was added and adjusted to pH 7.0. Finally, the pancreatin (2.4 mg/mL) and lipase (3.6 mg/mL) powders were added to the mixture and the timing started. The resulting mixture was incubated at 37 °C at 600 rpm for 2 h. During the digestion period, 0.1 M of the NaOH standard solution was added dropwise to maintain the pH at 7.0, and the consumption volume of NaOH at different digestion times was recorded. During simulated intestinal digestion, the lipids are broken down into two molecules of free fatty acids and one molecule of monoglycerides. Using the pH–stat method, the amount of total free fatty acids released in the simulated digestion was calculated according to the formula.

(2) $$\mathrm{Tatal}\;\mathrm{FFA}\;\mathrm{released}\;\left(\%\right)=\frac{C_{NaOH}\times V_{NaOH}\times 10^{-3}}{2\times \frac{m_{oil}}{M_{oil}}}\times 100\%$$

In the formula, *C_NaOH_* is the concentration of the NaOH standard solution (mol/L), *V_NaOH_* is the volume (mL) of the NaOH standard solution consumed at different digestion times (t), *m_oil_* is the mass of rapeseed oil contained in the digested emulsion (g), and *M_oil_* is the average molecular mass (g/mol) of rapeseed oil, which is 930 g/mol [26].

### 2.8. Measurement of Digestive Properties

The average particle size and ζ-potential of the samples were measured after the digestion system with different bile salt concentrations went through the various stages of simulated GIT. The average particle size (d_4,3_) is measured via a laser particle size analyzer MASTERSIZER 3000 (Malvern, Malvern City, UK), and the measurement method of ζ-potential is the same as method 2.7. To avoid the effect of multiple scattering, all samples were diluted with deionized water at the same pH.

### 2.9. Determination of UA Content via HPLC

HPLC determinations were performed using an Agilent 1100 LC system equipped with a quaternary solvent delivery system, autosampler, and diode array detector (DAD). The detection wavelength is 210 nm. The separation was performed on a YMC-Pack ODS-A (250 × 4.60 mm) column, and the oven and autosampler were kept at 30 °C. The mobile phase was HPLC grade methanol, the flow rate was 1 mL/min, the injection volume was 20 μL, and each sample was analyzed at least three times. A series of concentrations (20, 40, 80, 160, and 320 μg/mL) of the ursolic acid standard was prepared. Taking the concentration of ursolic acid as the abscissa and the peak area as the ordinate, the standard curve of ursolic acid was drawn. The linear regression equation is:

*A* = 0.8437 *m* + 4.1702 (3)

where *A* is the peak area of UA (mAUS), and *m* is the UA concentration (μg/mL). The correlation coefficient (R^2^) is 0.99743.

The UA content in the aqueous phase (micelles) of the digested samples was determined according to the method of Martin et al. [8], with slight modifications. At the end of digestion, the samples were centrifuged at 10,000 rpm for 10 min. After centrifugation, the intermediate micelles were collected using a syringe, where 1 mL of the sample was extracted from the micelles with 3 mL of anhydrous ethanol for 2 h at room temperature with stirring, left overnight, and then the supernatant was aspirated across the membrane (0.22 μm). The UA content in the micelles was determined via high-performance liquid chromatography, and the UA concentration in the aqueous phase of the sample could be calculated from the peak area and the standard curve of ursolic acid.

### 2.10. Statistical Analyses

All experiments were repeated three times, and the results were expressed as mean ± standard deviation. We used Excel 2016 (Microsoft, Redmond, WA, USA), PowerPoint 2016 (Microsoft, Redmond, WA, USA), and Origin 2022 (OriginLab, Northampton, MA, USA) for graph analysis.

## 3. Results

### 3.1. Effect of pH and Intestinal Salts on Emulsion Stability

The basic conditions of the intestinal digestion model are intestinal salts, pH, bile salts, and pancreatic enzymes [24]. Before exploring the role of the influence of bile salts, we investigated the properties of emulsions in the presence of only enteric salts to clarify whether intestinal pH and ionic strength would have any potential effect on emulsion stability. In general, the physiological ionic strength and the pH of the duodenum change significantly after a gastric emptying of food due to the food content, the presence of buffer salts, and the release of digestive products such as free fatty acids [29]. Therefore, in this part of the experiment, pH 6.0 and pH 7.5 were used to simulate the fasting and eating states, respectively. In addition, the pure water–emulsion control group without intestinal salt was set up, of which the pH was about 6.0.

#### 3.1.1. Appearance and Microstructure

**Figure 1:**
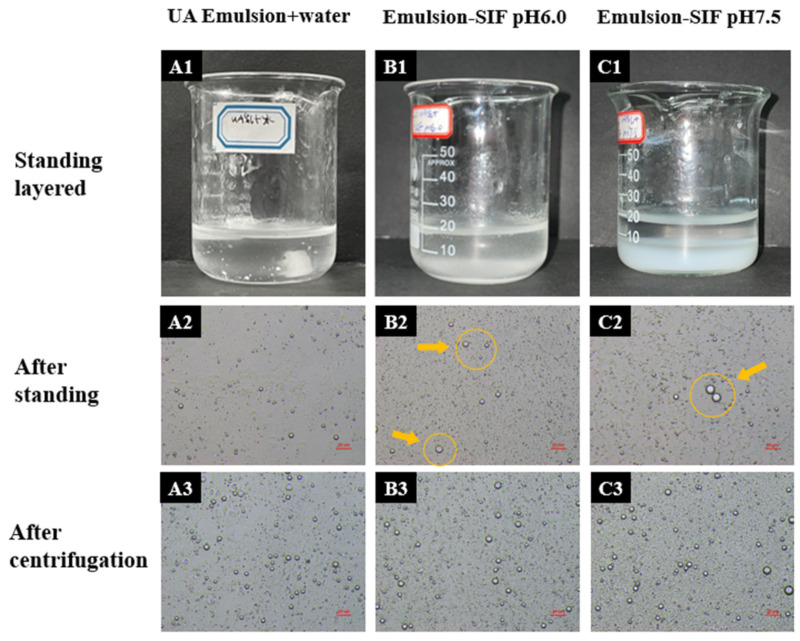
Appearance and microstructure images of UA emulsion + pure water pH 6.0 (**A1**–**A3**), UA emulsion + intestinal salt pH 6.0 (**B1**–**B3**), and UA emulsion + intestinal salt pH 7.5 (**C1**–**C3**). The arrows and circles in the image circle larger droplets of emulsion due to effects such as salt-induced aggregation.

Figure 1 shows the digested solution after partial simulated digestion (37.5 °C, 600 rpm, 2 h), which was left for a period to observe the change in emulsion morphology. It was found that the thickness of the emulsion layer in the pure water group was thinner, and some of the emulsion attached to the inner wall of the beaker could be observed. The digestive juice of the enteric salt group had obvious layers after standing; the upper layer was a thin emulsion, and there was no obvious difference to the naked eye; the middle layer was the water phase; and the bottom layer was the salt ion precipitation. Comparing Figure 1(B1,C2), it was found that the pH 7.5 system had significantly more white flocculent precipitates than the pH 6.0 mixed system, from which it can be concluded that pH affects the formation of salt precipitates such as Ca(OH)_2_, while the type and amount of salt ions affect the stability of the emulsion. It can be seen that the mechanisms of pH and ionic strength on emulsion stability are interrelated, and these two factors themselves also affect each other.

The upper emulsion of the digested solution was made into microscope slides after resting. Comparing the pH 6.0 intestinal salt group and the pH 6.0 pure water group, some larger droplets appeared at the interface (Figure 1); considering that salt ions have been mentioned in several studies to produce a salt-induced aggregation on the emulsion droplets, i.e., Ca^2+^, Na^+^, and other salt ions will cover and wrap around the droplet surface and even shield the droplets from mutual electrostatic interactions [25,30,31], it is presumed that the figure shows a similar phenomenon. The intestinal salt groups of pH 6.0 and pH 7.5 were compared with each other, and some larger droplets appeared in the microscopic images while other differences were not obvious.

Centrifugal stability can be used to simulate the state change in the samples after a long time of natural standing. It is a fast method to evaluate emulsion stability. The comparison of the images in the second and third rows shows that after centrifugation, part of the emulsion droplets increased significantly (Figure 1), but no demulsification and other phenomena occurred. It can be considered that the centrifugal stability of the UA emulsion is good, and the enteric salts and pH only accelerate the process of emulsion instability to some extent.

#### 3.1.2. Particle Size and ζ-Potential

**Figure 2:**
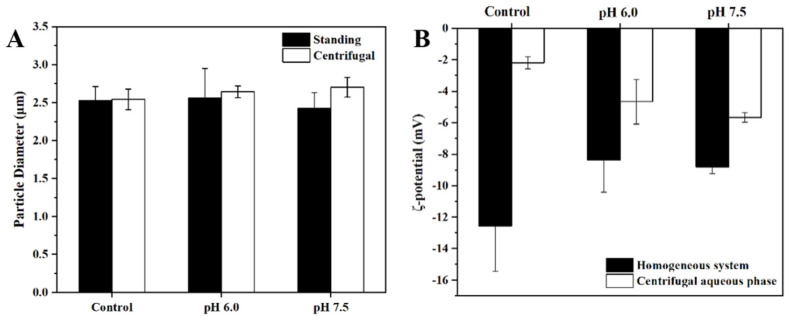
Emulsion + pure water pH 6.0, emulsion + intestinal salt pH 6.0, and emulsion + intestinal salt pH 7.5 after simulated digestion, static upper emulsion, and centrifugal emulsion particle size (**A**), and the ζ-potential value of homogeneous system and water phase after centrifugation of the three samples (**B**).

Particle size and ζ-potential results of the emulsion in pure water (pH 6.0) and the emulsion in the intestinal salt solution (pH 6.0 and 7.5, respectively) after simulated digestion are shown in Figure 2. There is no significant difference in particle size between the groups for both the upper emulsion after standing and the upper emulsion after centrifugation. The particle size of the centrifuged group would be higher than that of the resting group as a whole for the same reason as stated above. In comparison with the pH 6.0 enteric salt group, the pH 7.5 enteric salt group showed a larger change in particle size after centrifugation, further indicating that pH affects the centrifugal stability of the emulsion. The emulsion–pure water group at pH 6.0 and the emulsion–enteric salt group at pH 6.0 had no significant difference compared with each other, which represented that the enteric salt had little effect on the particle size and centrifugal stability of the emulsion.

ζ-potential reflects the electrostatic interaction between colloidal particles and can be used to determine the stability of the system. Generally speaking, the stability of the emulsion is proportional to the absolute value of the ζ-potential. It is known that the potential magnitude of the pure UA solid in the water phase is negative (−28.20 ± 0.78 mV), so the negative potential of the emulsion–pure water system is probably related to the part of UA released in the emulsion. Comparing it with the enteric salt group at pH 6.0, the absolute value of the uniform potential of the system decreases after the addition of salt ions, which may be related to the electrostatic repulsion between Na^+^, Ca^2+^, and other cations added. pH is one of the important factors affecting the ζ-potential, and the ζ-potential decreases with the increase in pH. Comparing the pH 6.0 intestinal salt group with the pH 7.5 intestinal salt group, there was no significant decrease in potential. It is speculated that more UA particles are released in the enteric salt system at pH 6.0. In general, pH and enteric salt both have a certain degree of influence on the stability of the emulsion, but the effect is not large.

### 3.2. Effect of Different Bile Salt Concentrations on Emulsion Stability

The analysis of the test results in the first part shows that the pH and intestinal salts have little effect on the stability of the emulsion, so we further investigated the effect of different bile salt concentrations on the characteristics of the emulsion by keeping the pH and ionic strength constant without the addition of pancreatic enzymes. Since the ingestion of the emulsion is in the fed state and the intestinal pH fluctuates between 6~7.5, the pH was chosen to be fixed at 7.0 for this part of the experiment [25]. In addition, the concentration of bile salts in the intestine varies depending on the feeding state and food components ingested [20]. To comprehensively consider all cases, a concentration gradient of 0 to 25 mg/mL was selected for the experiment.

#### 3.2.1. Appearance and Microstructure

**Figure 3:**
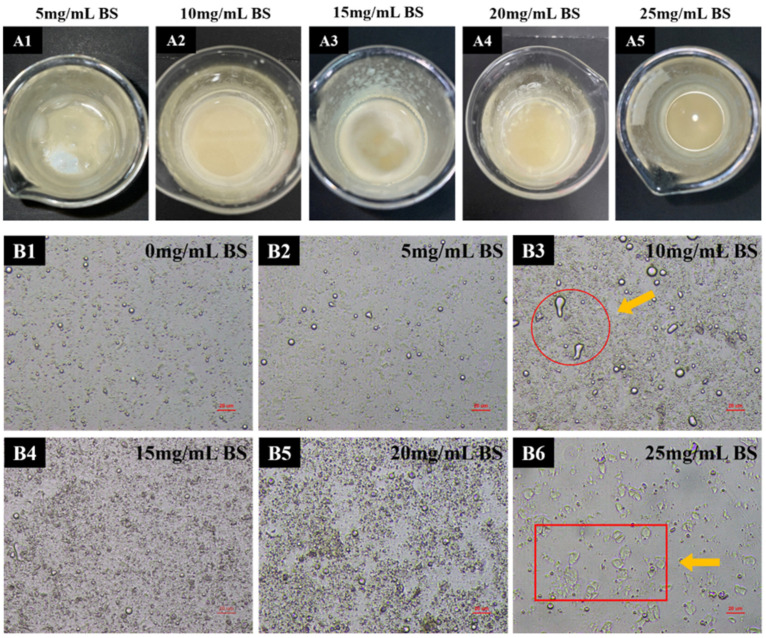
Appearance (**A1**–**A5**) and microstructure (**B1**–**B6**) of emulsions as a function of bile salt concentration in a simulated digestive system without the addition of pancreatic enzymes. The arrows and circles of (**B3**) are deformed droplets. Due to the increased concentration of bile salts, the droplets of the emulsion change from round to irregular. The arrows and rectangles in (**B6**) circle some ursolic acid fragments. Due to the high concentration of bile salts, the droplets of the emulsion are broken, and some ursolic acid fragments are also displaced.

The appearance and microstructure of the emulsion after the static simulated digestion conditions (without enzymes) at different bile salt concentrations is shown in Figure 3. When the bile salt concentration is 5 mg/mL, the upper layer of the digest was visible as an oil-like emulsion (Figure 3(A1)). When the bile salt concentration was 10 mg/mL, the upper layer became a white mixture of oil and flocculent (Figure 3(A2)). When the bile salt concentration was 15 mg/mL and 20 mg/mL, the upper layer of the digest was completely white flocculent. And, by the time the concentration was 25 mg/mL, there was no visible upper layer material (Figure 3(A5)). In summary, bile salts had a large effect on the appearance of UA emulsions.

As shown in Figure 3(B2), when the bile salt concentration is 5 mg/mL, the complete emulsion droplets are visible in the field of view. When the bile salt concentration is 10 mg/mL, some droplets in the interface become larger in diameter and appeared as clearly deformed droplets. Since the concentration of bile salt added at this time is much higher than the critical micelle concentration (CMC), a large number of bile salt micelles are self-assembled in the continuous phase. These micelles are squeezed from the space of the emulsion drops, which brings a depleting effect to the system. This entropy force brings the droplets closer to each other and clumps together to form larger particles [32]. On the other hand, the hydrophobic binding of bile salts to the UA molecules at the interface destroys the Pickering stabilization mechanism of UA crystals, resulting in the surface deformation of droplets and the loss of emulsion stability. Combined with the appearance morphology at the concentration of 10 mg/mL in Figure 3(A2), the emulsion slowly changes from oil-like to white flocculent from this concentration.

When the bile salt concentration was 15 mg/mL and 20 mg/mL, the droplet size in the field of view slowly became smaller. At this point, the emulsion morphology changed to a white flocculent form, and the original size droplets were broken down into smaller droplets. More precipitates in the background may be crystallites formed by the bile salts and Ca^2+^. When the bile salt concentration was 25 mg/mL, the droplet size became smaller and massive crystals appeared at the interface as UA crystals. So, it can be clearly seen from Figure 3B that the droplet size continues to decrease with the increase in bile salt concentration. The salt-induced aggregates that appeared at the previous interface ruptured [33,34,35].

#### 3.2.2. Particle Size and ζ-Potential

**Figure 4:**
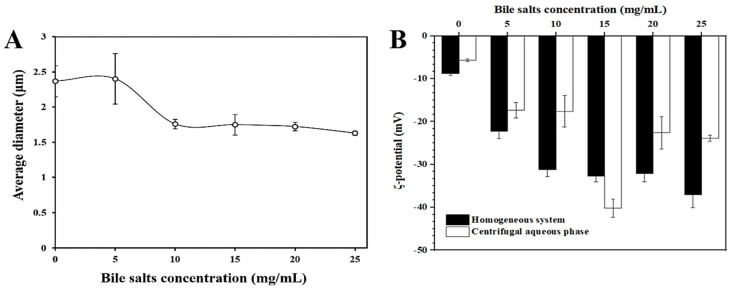
Emulsion particle size (**A**) and ζ-potential (**B**) changes of emulsion homogeneous system and centrifugal aqueous phase under static simulated digestion system (without pancreatic enzyme) with different bile salt concentrations.

Figure 4A shows the average particle size of the UA emulsion in the presence of different bile salt concentrations. Initially, the droplet size was about 2.37 ± 0.22 μm. When the bile salt concentration was 5 mg/mL, a slight increase in the mean size of the emulsion was negligible. When the amount of bile salt added was higher than 5 mg/mL, the average particle size decreased significantly and did not decrease further after reaching a relatively constant low value. This indicates that there is a certain interaction between the UA-stabilized emulsion droplets and bile salts. The addition of more bile salts resulted in a decrease in the apparent size of the droplets, possibly because the previously formed salt-induced aggregates break up [25]. Due to the salt-induced effect, the charge on the surface of the emulsion droplet is unstable, which is easy to agglomerate and form droplets with a larger particle size. However, the addition of bile salt will affect or even destroy the previous salt-induced aggregates, and the larger droplet will disperse and become a small droplet. For another, bile salts bind to the UA crystals on the interface via hydrophobic interaction, or replace UA with a continuous phase, breaking its stability mechanism for the W/O emulsion so the droplets break up and the size of the system decreases.

Figure 4B shows the ζ-potential of the digested system for different bile salt concentrations. As the bile salt concentration increased from 0.0 mg/mL to 25.0 mg/mL, the ζ-potential of the homogeneous system also decreased substantially from −8.81 ± 0.42 mV to −37.10 ± 3.04 mV. The change in the overall potential is related to the addition of the bile salts. The polar surface of bile salts contains negatively charged -COOH groups [36], which will reduce the potential of the system. And also, possibly due to the displacement of UA crystals at the oil–water interface [37,38]. During the whole experiment, the ζ-potential of the system did not reach the zeta-potential of supersaturated bile salt droplets (−51.3 ± 2.5 mV) [25], which indicates that there must be some interaction between the bile salts and the droplet that makes the UA neither completely displaced nor secondarily covered by the bile salt.

### 3.3. Effect of Different Bile Salt Concentrations on the In Vitro Simulated Digestion of Emulsion

After the above static in vitro simulated digestion test (without enzymes), we have confirmed that bile salts have a certain effect on the properties of the emulsion. In the following experiments, 2.4 mg/mL pancreatin and 3.6 mg/mL lipase will be added to more realistically simulate in vitro oral–gastric–gut digestion. Different bile salt concentrations of 0.0, 1.0, 2.0, 5.0, 10.0, and 15.0 mg/mL were also selected to further investigate the effect of bile salts on the digestion of the emulsion and UA bioaccessibility by observing the appearance of the digests, measuring the particle size and ζ-potential of the samples, and calculating the UA concentration in the aqueous phase.

#### 3.3.1. Appearance

**Figure 5:**
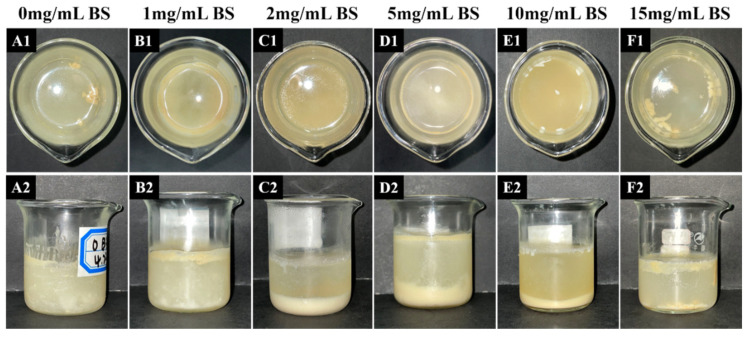
The appearance of the emulsion after digestion is simulated in vitro with different bile salt concentrations. The image on the top line is a top view of the digestive juice, which allows a clearer view of the floating emulsion in the upper layer. The image below is a front view of the digestive juice, which allows a clearer view of the layering phenomenon.

After the UA emulsion was digested in the oral, gastric, and intestinal phases and was left to stand for one day, the appearance was photographed. The results are shown in Figure 5. All samples showed a clear layering phenomenon, where the upper layer was the undigested emulsion, the middle layer was the water phase, and the lower layer was the salt ion precipitation. From Figure 5(A1), it is clear that the emulsions were poorly digested when bile salts were not added. When the bile salt concentration was less than 5 mg/mL, the upper emulsion was digested relatively cleanly, and no obvious emulsion was observed. Compared to the samples without the added bile salts, it can be shown that the low level of bile salts facilitates the digestion of the emulsion. When the bile salt concentration was higher than 10 mg/mL, the digestibility of the emulsion turned down. It can be seen that the high concentration of bile salts did not further promote the digestion of the emulsion, but inhibited lipolysis.

#### 3.3.2. Particle Size and ζ-Potential

**Figure 6:**
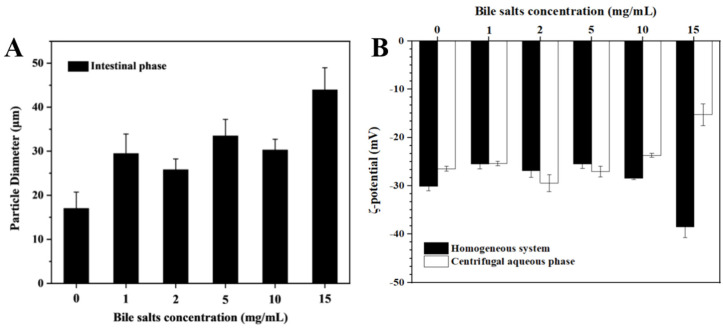
The particle size (**A**) and ζ-potential (**B**) of the emulsion after digestion is simulated in vitro with different bile salt concentrations.

**Figure 7:**
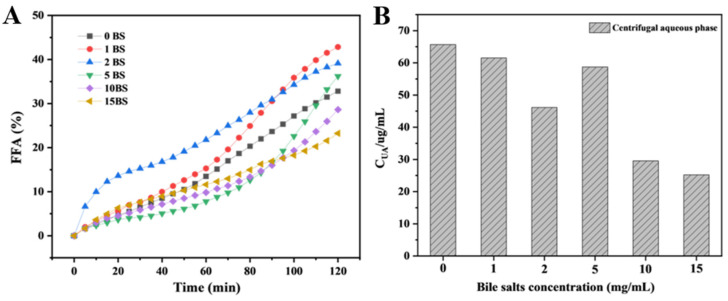
The free fatty acid release rate (**A**) and the UA concentration in the aqueous phase of the digestive fluid (**B**) during emulsion digestion at different bile salt concentrations.

Figure 6A shows the changes in the particle size of the emulsions after in vitro simulated digestion with different bile salt concentrations. The digestive solution without bile salts has a smaller particle size. The reason may be that many emulsions are undigested, flocculent, lumpy, and are not uniformly mixed in the entire system. The particle size was relatively small for the bile salt concentrations of 1 mg/mL and 2 mg/mL, which is also consistent with the FFA release curve in Figure 7. Due to the higher degree of digestion of the emulsion, the larger droplets in the system are decomposed into small molecular particles. When the bile salt was added at 5 mg/mL, the amount and rate of the FFA release were reduced, so the particle size was larger. When the bile salt concentration was 10 mg/mL, its particle size should be larger according to the FFA release curve, but after measurement, its particle size was smaller than the sample group of 5 mg/mL instead. This may be because the oil–water interface was filled with bile salt molecules and the UA crystals were forced to be displaced and recombined into small droplets via bile salt wrapping. As expected, when the bile salt concentration reached 15 mg/mL, the particle size was the highest, reaching 38.2 ± 5.02 μm. This is because the high concentration of bile salts inhibits the decomposition of lipase.

Figure 6B shows the potential magnitudes in the simulated digestion system in vitro at different bile salt concentrations. After adding a certain amount of bile salts, the potential has been maintained at a relatively stable −28 mV, which is very close to the potential of the pure UA solid in the aqueous phase (−28.20 ± 0.78 mV). Combined with the digestion curve in Figure 7 and the particle size in Figure 6A, this situation is likely to be related to the replacement of bile salts. After the bile salts were adsorbed to the oil–water interface, the UA crystals were replaced and squeezed into the continuous phase [20]. Some studies have also shown that the significantly negative ζ-potential during intestinal digestion may be due to the release of free fatty acids or the presence of anionic species such as hydroxyl ions [39]. When the bile salt concentration was increased to 15 mg/mL, there was a small decrease in the ζ-potential. This is related to excessive bile salt addition. The remaining free bile salt molecules dissolve in the water phase, and their -COOH reduces the potential.

#### 3.3.3. Free Fatty Acid Release Rate (FFA%) and Bioaccessibility

The FFA release rate of the emulsion in the external simulated digestion system with different bile salt concentrations is shown in Figure 7A. From the figure, when the concentration is 1 mg/mL and 2 mg/mL, the release amount of FFA is relatively high, reaching 42.83% and 39.13%, respectively. This is consistent with the conclusions of other literature, which stated that the low concentration of bile salt can promote the digestion via various mechanisms. When the addition of bile salts increased to 5 mg/mL, although the total FFA release was not low, the rate was very small during the first 1 h of the simulated digestion. This is related to the adsorption and desorption behavior of bile salts [40]. The interface behavior during lipid digestion is relatively complex: firstly, bile salts are adsorbed to the oil–water interface, and then they interact electrostatically with colipase to help the lipase bind to the oil–water interface dominated by the bile salts [38]. In addition, some of the early production of lipolysis products further accumulated on the interface, so the digestion rate in the early stage is very slow. When the addition of bile salts increased above 10 mg/mL, both the rate of the FFA release and the total FFA release was significantly reduced. At this time, excessive bile salts covered the interface twice, occupying the binding site of the enzyme and the space where the lipolysis reaction occurs.

The UA concentrations in the aqueous phase of the digestive juice under different bile salt concentrations are shown in Figure 7, which can be used for the rough analysis of the bioaccessibility of UA. It is known that the oil solubility of UA is better, and when the bile salt concentration is 0 mg/mL, the highest concentration of UA is present in the system. This indicates that, during the simulated digestion, many factors will affect the stability of the UA emulsion, resulting in the release of UA. After adding a little bile salt, the concentration of UA in the aqueous phase remained high, which was just in line with our previous hypothesis that bile salts displaced UA at the interface, which was conducive to lipase adsorption. At the same time, the UA displacement also promoted the instability process of the emulsion and accelerated the decomposition and utilization of lipase to the oil phase. As the bile salt concentration continued to increase, the release rate and the total level of free fatty acids in the emulsion decreased, and the UA content in the continuous phase also decreased. The depleted flocculation caused by the formation of a large number of bile salt micelles in the continuous phase forces UA in the water phase to adsorb to the interface to maintain the stability of the emulsion, and bile salt binds to the interface via hydrophobic action to cover the UA crystals twice.

## 4. Conclusions

This study explored the effects of bile salts on UA’s emulsion stability and digestion characteristics. The results show that basic factors such as pH and intestinal salt had little effect on the emulsion itself. In the static simulation of small intestine digestion, bile salts can significantly affect the stability of the emulsion. With the increase in bile salt concentration, the emulsion morphology changes, the stability decreases, and the particle size also drops, which could be caused by the emulsification of bile salt and the hydrophobic interaction with UA. In dynamic oral–gastric–gut in vitro simulated digestion, the addition of bile salts can significantly affect the digestibility of emulsions by changing the degree of hydrolysis and the rate of the FFA release. Low concentrations of bile salts can promote emulsion digestion via displacement, while high concentrations of bile salts inhibit emulsion digestion due to the secondary coverage. Understanding the stability and digestion of the UA emulsion in the human body provides a theoretical basis for expanding the practical application of the UA emulsion and also gives information for improving the bioavailability of hydrophobic bioactive compounds.

## Data Availability

The data are contained within the article.

## References

[1] Ma J.-Q., Ding J., Zhang L., Liu C.-M. (2014). Ursolic acid protects mouse liver against CCl 4 -induced oxidative stress and inflammation by the MAPK/NF-κB pathway. Environ. Toxicol. Pharmacol..

[2] Lu Y., Kan H., Wang Y., Wang D., Wang X., Gao J., Zhu L. (2018). Asiatic acid ameliorates hepatic ischemia/reperfusion injury in rats via mitochondria-targeted protective mechanism. Toxicol. Appl. Pharmacol..

[3] Jamkhande P.G., Pathan S.K., Wadher S.J. (2016). In silico PASS analysis and determination of antimycobacterial, antifungal, and antioxidant efficacies of maslinic acid in an extract rich in pentacyclic triterpenoids. Int. J. Mycobacteriol..

[4] Kaps A., Gwiazdon P., Chodurek E. (2021). Nanoformulations for Delivery of Pentacyclic Triterpenoids in Anticancer Therapies. Molecules.

[5] Valdes K., Morales J., Rodriguez L., Gunther G. (2016). Potential use of nanocarriers with pentacyclic triterpenes in cancer treatments. Nanomedicine.

[6] Zhong Y., Liang N., Liu Y., Cheng M.-S. (2021). Recent progress on betulinic acid and its derivatives as antitumor agents: A mini review. Chin. J. Nat. Med..

[7] Furtado N.A.J.C., Pirson L., Edelberg H., Miranda L.M., Loira-Pastoriza C., Preat V., Larondelle Y., Andre C.M. (2017). Pentacyclic Triterpene Bioavailability: An Overview of In Vitro and In Vivo Studies. Molecules.

[8] Martin D., Navarro del Hierro J., Villanueva Bermejo D., Fernandez-Ruiz R., Fornari T., Reglero G. (2016). Bioaccessibility and Antioxidant Activity of Calendula officinalis Supercritical Extract as Affected by in Vitro Codigestion with Olive Oil. J. Agric. Food Chem..

[9] McClements D.J., Zou L., Zhang R., Salvia-Trujillo L., Kumosani T., Xiao H. (2015). Enhancing Nutraceutical Performance Using Excipient Foods: Designing Food Structures and Compositions to Increase Bioavailability. Compr. Rev. Food Sci. Food Saf..

[10] Zheng L., Cao C., Chen Z., Cao L., Huang Q., Song B. (2020). Evaluation of emulsion stability by monitoring the interaction between droplets. Lwt-Food Sci. Technol..

[11] Aboalnaja K.O., Yaghmoor S., Kumosani T.A., McClements D.J. (2016). Utilization of nanoemulsions to enhance bioactivity of pharmaceuticals, supplements, and nutraceuticals: Nanoemulsion delivery systems and nanoemulsion excipient systems. Expert Opin. Drug Deliv..

[12] Ozturk B., Argin S., Ozilgen M., McClements D.J. (2015). Nanoemulsion delivery systems for oil-soluble vitamins: Influence of carrier oil type on lipid digestion and vitamin D-3 bioaccessibility. Food Chem..

[13] Davidov-Pardo G., McClements D.J. (2015). Nutraceutical delivery systems: Resveratrol encapsulation in grape seed oil nanoemulsions formed by spontaneous emulsification. Food Chem..

[14] Danielsen E.M., Hansen G.H., Rasmussen K., Niels-Christiansen L.-L. (2013). Permeabilization of enterocytes induced by absorption of dietary fat. Mol. Membr. Biol..

[15] Gupta S., Kesarla R., Omri A. (2013). Formulation strategies to improve the bioavailability of poorly absorbed drugs with special emphasis on self-emulsifying systems. ISRN Pharm..

[16] Bauer E., Jakob S., Mosenthin R. (2005). Principles of Physiology of Lipid Digestion. Asian-Australas. J. Anim. Sci..

[17] McClements D.J., Decker E.A., Park Y., Weiss J. (2008). Designing food structure to control stability, digestion, release and absorption of lipophilic food components. Food Biophys..

[18] Reis P., Holmberg K., Watzke H., Leser M.E., Miller R. (2009). Lipases at interfaces: A review. Adv. Colloid Interface Sci..

[19] Reis P., Watzke H., Leser M., Holmberg K., Miller R. (2010). Interfacial mechanism of lipolysis as self-regulated process. Biophys. Chem..

[20] Maldonado-Valderrama J., Wilde P., Macierzanka A., Mackie A. (2011). The role of bile salts in digestion. Adv. Colloid Interface Sci..

[21] Macierzanka A., Torcello-Gomez A., Jungnickel C., Maldonado-Valderrama J. (2019). Bile salts in digestion and transport of lipids. Adv. Colloid Interface Sci..

[22] Liu Y.G., Xia H.P., Guo S.Y., Lu X.Y., Zeng C.X. (2022). Development and characterization of a novel naturally occurring pentacyclic triterpene self-stabilized pickering emulsion. Colloids Surf. A-Physicochem. Eng. Asp..

[23] Liu S.Q., Liu H., Zhang L.L., Ma C., Abd El-Aty A.M. (2022). Edible pentacyclic triterpenes: A review of their sources, bioactivities, bioavailability, self-assembly behavior, and emerging applications as functional delivery vehicles. Crit. Rev. Food Sci. Nutr..

[24] Qian C., Decker E.A., Xiao H., McClements D.J. (2012). Nanoemulsion delivery systems: Influence of carrier oil on beta-carotene bioaccessibility. Food Chem..

[25] Sarkar A., Horne D.S., Singh H. (2010). Interactions of milk protein-stabilized oil-in-water emulsions with bile salts in a simulated upper intestinal model. Food Hydrocoll..

[26] Zhan Y., Chen C., Yuanfa L., Peirang C., Qiu L. (2018). Digestion fates of different edible oils vary with their composition specificities and interactions with bile salts. Food Res. Int..

[27] Ojeda-Serna I.E., Rocha-Guzman N.E., Gallegos-Infante J.A., Chairez-Ramirez M.H., Rosas-Flores W., Perez-Martinez J.D., Moreno-Jimenez M.R., Gonzalez-Laredo R.F. (2019). Water-in-oil organogel based emulsions as a tool for increasing bioaccessibility and cell permeability of poorly water-soluble nutraceuticals. Food Res. Int..

[28] Ma J.-J., Huang X.-N., Yin S.-W., Yu Y.-G., Yang X.-Q. (2021). Bioavailability of quercetin in zein-based colloidal particles-stabilized Pickering emulsions investigated by the in vitro digestion coupled with Caco-2 cell monolayer model. Food Chem..

[29] Kalantzi L., Goumas K., Kalioras V., Abrahamsson B., Dressman J.B., Reppas C. (2006). Characterization of the human upper gastrointestinal contents under conditions simulating bioavailability/bioequivalence studies. Pharm. Res..

[30] Guzey D., Kim H.J., McClements D.J. (2004). Factors influencing the production of o/w emulsions stabilized by beta-lactoglobulin-pectin membranes. Food Hydrocoll..

[31] Kulmyrzaev A., Chanamai R., McClements D.J. (2000). Influence of pH and CaCl_2_ on the stability of dilute whey protein stabilized emulsions. Food Res. Int..

[32] Jódar-Reyes A.B., Torcello-Gómez A., Wulff-Pérez M., Gálvez-Ruiz M.J., Martín-Rodríguez A. (2010). Different stability regimes of oil-in-water emulsions in the presence of bile salts. Food Res. Int..

[33] Morris V.J., Gunning A.P. (2008). Microscopy, microstructure and displacement of proteins from interfaces: Implications for food quality and digestion. Soft Matter.

[34] Maldonado-Valderrama J., Woodward N.C., Gunning A.P., Ridout M.J., Husband F.A., Mackie A.R., Morris V.J., Wilde P.J. (2008). Interfacial characterization of beta-lactoglobulin networks: Displacement by bile salts. Langmuir.

[35] Winuprasith T., Chantarak S., Suphantharika M., He L., McClements D.J. (2014). Alterations in nanoparticle protein corona by biological surfactants: Impact of bile salts on beta-lactoglobulin-coated gold nanoparticles. J. Colloid Interface Sci..

[36] Parker R., Rigby N.M., Ridout M.J., Gunning A.P., Wilde P.J. (2014). The adsorption-desorption behaviour and structure function relationships of bile salts. Soft Matter.

[37] Mun S., Decker E.A., McClements D.J. (2007). Influence of emulsifier type on in vitro digestibility of lipid droplets by pancreatic lipase. Food Res. Int..

[38] Euston S.R., Baird W.G., Campbell L., Kuhns M. (2013). Competitive Adsorption of Dihydroxy and Trihydroxy Bile Salts with Whey Protein and Casein in Oil-in-Water Emulsions. Biomacromolecules.

[39] Yunbing T., Zhiyun Z., Hualu Z., Hang X., McClements D.J. (2020). Factors impacting lipid digestion and beta-carotene bioaccessibility assessed by standardized gastrointestinal model (INFOGEST): Oil droplet concentration. Food Funct..

[40] Euston S.R., Bellstedt U., Schillbach K., Hughes P.S. (2011). The adsorption and competitive adsorption of bile salts and whey protein at the oil-water interface. Soft Matter.

